# Factors associated with non-adherence to highly active antiretroviral therapy in Nairobi, Kenya

**DOI:** 10.1186/1742-6405-8-43

**Published:** 2011-12-05

**Authors:** Samwel N Wakibi, Zipporah W Ng'ang'a, Gabriel G Mbugua

**Affiliations:** 1 Institute of Tropical Medicine and Infectious Diseases (ITROMID), Jomo Kenyatta University of Agriculture and Technology (JKUAT), P.O. Box 62000 - 00200 Nairobi, Kenya; 2 Centre for Microbiology Research, Kenya Medical Research Institute (KEMRI), P.O Box 19464 00200 Nairobi, Kenya

## Abstract

**Background:**

Antiretroviral therapy (ART) requires high-level (> 95%) adherence. Kenya is rolling out ART access programmes and, issue of adherence to therapy is therefore imperative. However, published data on adherence to ART in Kenya is limited. This study assessed adherence to ART and identified factors responsible for non adherence in Nairobi.

**Methods:**

This is a multiple facility-based cross-sectional study, where 416 patients aged over 18 years were systematically selected and interviewed using a structured questionnaire about their experience taking ART. Additional data was extracted from hospital records. Patients were grouped into adherent and non-adherent based on a composite score derived from a three questions adherence tool developed by Center for Adherence Support Evaluation (CASE). Multivariate regression model was used to determine predictors of non-adherence.

**Results:**

Overall, 403 patients responded; 35% males and 65% females, 18% were non-adherent, and main (38%) reason for missing therapy were being busy and forgetting. Accessing ART in a clinic within walking distance from home (OR = 2.387, CI._95 _= 1.155-4.931; *p = *0.019) and difficulty with dosing schedule (OR = 2.310, CI._95 _= 1.211-4.408, *p = *0.011) predicted non-adherence.

**Conclusions:**

The study found better adherence to HAART in Nairobi compared to previous studies in Kenya. However, this can be improved further by employing fitting strategies to improve patients' ability to fit therapy in own lifestyle and cue-dose training to impact forgetfulness. Further work to determine why patients accessing therapy from ARV clinics within walking distance from their residence did not adhere is recommended.

## Background

Antiretroviral treatment success depends on sustainable high rates of adherence to medication regimen of ART [1]. However, significant proportions of HIV-infected patients do not reach high levels of adherence and this can lead to devastating public health problems. Mills *et al *in a meta-analysis study found a combined continental adherence to ART of 64% with 55% adherence in North America and 77% in Africa. Twenty four percent non-adherence has been reported in Southwest Ethiopia [2], 22% in Cote d'Ivore [3] and 13% in Cameroon [4]. Byakika *et al *[5] reported 68% adherence to HIV treatment in Uganda, 54% in Nigeria [6] and 63% in South Africa [7]. Non-adherence to ART has been associated to diverse factors including patient related factors, health condition/disease, health care system and healthcare teams, therapy/treatment and Socio-economic factors [5,6,8-10]. Kenya is rolling out a free HAART programme to increase access and by 2009, 336980 patients were accessing [11], and adherence reported vary from 48% in Kibera, Nairobi [12]; 56.8% in Eldoret [13] and 64% in Mombasa [14].

### Statement of the research hypothesis

Published data about factors that influence non-adherence to ART in Kenya is limited. To generate this knowledge, this study determined prevalence of non-adherence and associated factors.

It was hypothesized that:

H_0 _- there are no factors association with non-adherence to HAART among HIV patients in Nairobi, Kenya.

## Method

### Setting

This study was conducted at HIV/AIDS treatment centre in the Kenyatta National Hospital, Kenya Medical Research Institute (KEMRI) and Riruta Health centre in Nairobi, Kenya.

### Population sample

The sample was of 416 HIV+ outpatients aged 18 years or more on free HAART for three or more months between November, 2008 and April, 2009.

### Design

Cross-sectional observational study design collected data from systematically selected patients as they visited the three purposively selected HIV clinics to refill. The patients were interviewed about their health beliefs, health system interaction, ARV therapy uptake and reasons for non-adherence. CASE adherence tool [15] was used to determine adherence. Additional data about time on ARV and age were extracted from hospital records.

CASE Adherence tool was developed by the New York Academy of Medicine's (NYAM) Center for Adherence Support Evaluation (CASE). It consists of three adherence questions: (rated on a likert scale) "How often do you feel that you have difficulty taking your HIV medications on time? On average, how many days PER WEEK would you say that you missed at least one dose of your HIV medications? When was the last time you missed at least one dose of your HIV medications?"A higher composite score signify better adherence.

The CASE Adherence Index correlated strongly with the AACTG three-day self-reported adherence data (*p *< 0.001), is more strongly associated with HIV outcomes and performed as well as the three-day self-report when predicting CD4 cell count status[16].

### Data analysis

Patient's scores in the CASE adherence tool were summed up to obtain a composite score that ranged from 3 to 16 points. Patients with Index score ≤10 points were classified as non-adherent and > 10 adherent [15]. Other data generated from the questionnaire were keyed into SPSS and analyzed for frequencies, cross-tabulations, chi-square test, and multivariate logistic regression to determine predicting factors. A *p-value *< 0.05 was considered significant in all statistical analysis.

### Ethical considerations

Ethical clearance was given by the KEMRI's National Ethical Review Committee, and permission to conduct the research was obtained from the participating clinics. Consent was obtained both verbally and in writing in either English or Kiswahili. To ensure confidentiality, interviews were conducted in private and strict control maintained over data.

## Results

### Characteristics of the respondents

Characteristics of the respondents are presented in Table 1. Out of the 416 patients interviewed, 403 answered all adherence questions and were analyzed. Females were 262 (65%) and males 141 (35%); their age ranged between 18 and 64 years old. Most patients were married 213 (53%); 246 (61%) had at least secondary education, 145 (36%) had primary education and 10 (3%) had no formal education. Three hundred (75%) respondents earned less than $120 per month; and most 271(67%) lived in 1-3 rooms and the rest in a more than 1 bedroom house.

**Table 1:** Characteristics of respondents

Characteristics	Adherent			*p*-value
**Variable**	**No; *n (%)***		**Total; *n (%)***	

	**Socio-demographic characteristics**			

*Gender distribution *				
All	72 (18)	331 (82)	403 (100)	
Males	25 (18)	116 (82)	141 (35)	0.958
Females	47 (18)	215 (82)	262 (65)	

*Mean age*				
All	37.6 yrs	40.2 yrs	39.7 yrs	0.017*
Male	41.6 yrs	42.8 yrs	42.6 yrs	0.491
Female	35.4 yrs	38.8 yrs	38.2 yrs	0.012*

*Marital status *				0.225
Never married	14 (19)	58 (81)	72 (18)	
Married	40 (19)	173 (81)	213 (53)	
Divorced/Separate	13 (23)	44 (77)	57 (14)	
Widowed	5 (9)	52 (91)	57 (14)	
Missing	0	4	4 (1)	
*Ever widowed?*				0.053
Yes	5 (9)	52 (91)	57 (14)	
No	67 (20)	275 (80)	342 (85)	

*Education level*				0.665
*No education*	*1 (10)*	*9 (10)*	10 (3)	
*Primary*	*28 (19)*	*117 (81)*	145(36)	
*Secondary*	*32 (16)*	*168 (84)*	200(50)	
*Post secondary*	*10 (22)*	*36 (78)*	46 (11)	
*Missing*			2	

*Monthly Income (USD)*				0.734
*Unemployed*	*27 (20)*	*110 (80)*	137(34)	
*< 60*	*17 (21)*	*66 (80)*	83 (21)	
*61 - 120*	*11 (14)*	*69 (86)*	80 (20)	
*121 - 180*	*2 (9)*	*21 (91)*	23 (6)	
*181 - 240*	*5 (19)*	*22 (82)*	27 (7)	
*241 - 600*	*5 (15)*	*29 (85)*	34 (8)	
*600+*	*4 (24)*	*13 (77)*	17 (4)	
*Missing*			2	

*Housing*				0.275
*1 room*	*39 (21)*	*147 (79)*	186(46)	
*2-3 rooms*	*16 (19)*	*69 (81)*	85 (21)	
*1 bedroom*	*7 (17)*	*34 (83)*	41 (10)	
*2 bedrooms*	*4 (8)*	*48 (92)*	52 (13)	
*≥ 3 bedrooms*	*6 (15)*	*33 (85)*	39 (10)	
				
*1-3 rooms*	*55 (20)*	*216 (80)*	271 (67)	0.068
*≥ 1 bedrooms*	*17 (13)*	*115 (87)*	132 (33)	

	***Clinical characteristics***			

*CD4 cell count ≤200 cells/ml*				0.025*
*Yes*	*25(26)*	*71 (74)*	96 (24)	
*No*	*44(16)*	*237(84)*	281(70)	
*Missing*			26 (6)	

*Time on ART *				0.003*
*3 to 6 months*	*13(37)*	*22 (63)*	35 (9)	
*6 - 12 months *	*18(21)*	*69 (79)*	87 (22)	
*1 to 2 years*	*25(19)*	*110(81)*	135(34)	
*3 years +*	*16(11)*	*129(89)*	145(36)	
*Missing*			1	

*Adverse effects *				
*Nausea*	*7 (30)*	*16 (70)*	23 (7)	0.898
*Vomiting*	*1 (8)*	*11 (92)*	12 (4)	
*Diarrhea*	*3 (23)*	*10 (77)*	13 (4)	
*Neuropathy*	*3 (10)*	*27 (90)*	30 (9)	
*Itching*	*6 (23)*	*20 (77)*	26 (8)	
*Rashes*	*5 (12)*	*36 (88)*	41 (13)	
*Others*	*6 (15)*	*33 (85)*	39 (12)	
*None*	*24(17)*	*114(83)*	138(43)	

	**ART knowledge**			

*Health literacy*				
*Adhere*	*57 (17)*	*272 (83)*	329 (82)	0.55
*Condom use*	*35 (18)*	*162 (82)*	197 (49)	0.959
*Nutrition*	*43 (16)*	*233 (84)*	276 (68)	0.077
				
*Use*				
*Identify drugs*	*72 (18)*	*331 (82)*	403 (100)	
*Dosage*	*72 (18)*	*330 (82)*	402 (100)	
*Life-long therapy*	*72 (18)*	*328 (82)*	400 (99)	

	**Social support**			

*Living with*				0.484
*Family*	*53(17)*	*268(83)*	321(80)	
*Friends*	*2 (22)*	*7 (78)*	9 (2)	
*Alone*	*16(22)*	*56 (78)*	72 (18)	

*Living with children?*				0.345
*No*	*10(14)*	*62 (86)*	72 (18)	
*Yes *	*50(18)*	*235(82)*	285(71)	
*Have no children*	*12(26)*	*34 (74)*	46 (11)	0.122
*Has children*	*60(17)*	*297(83)*	357(89)	

*HIV Status disclosed*				0.794
*Yes:*	*58(18)*	*271(82)*	329(82)	
*No: *	*13(18)*	*60 (81)*	73 (18)	

*Reminded dose by *				0.781
*Self*	*36(19)*	*151(81)*	187(46)	
*Family*	*35(17)*	*176(83)*	211(52)	
*Friends *	*1 (20)*	*4 (80)*	5 (1)	

*Level of social Support *				
*Never (0%)*	*13(23)*	*43 (77)*	56 (14)	
*Rarely (25%)*	*5 (28)*	*13 (72)*	18 (5)	
*Sometimes (50%)*	*12(26)*	*34 (74)*	46 (11)	
*Often (75%)*	*17(22)*	*60 (78)*	77 (19)	
*Always (> 75%)*	*24(18)*	*181(88)*	205(51)	
*Missing*			1	
*Felt social Support*				0.030*
*< Sometimes*	*18(24)*	*56 (76)*	74 (21)	
*> Sometimes*	*41(14)*	*241(86)*	282(79)	

	***Concerns about therapy***			

*Taking ART worries me*				0.378
*Agree*	*16 (22)*	*58 (78)*	74 (18)	
*Disagree*	*55 (17)*	*264 (83)*	319 (79)	
*Not sure*			10 (3)	
*I Worry about long-term effects of ART*				0.885
*Agree*	*31 (19)*	*135 (81)*	166 (41)	
*Disagree*	*40 (18)*	*181 (82)*	221 (55)	
*Not sure*			16 (4)	
*Lack understanding of how ART works *				0.505
*Agree*	*28 (20)*	*115 (80)*	143 (35)	
*Disagree*	*40 (17)*	*197 (83)*	237 (59)	
*Not sure*			23 (6)	
*Therapy disrupt my life*				0.006*
*Agree*	*20 (29)*	*49 (71)*	69 (17)	
*Disagree*	*49 (15)*	*274 (85)*	323 (80)	
*Not sure*			11 (3)	
*I am embarrassed taking ARV*				0.162
*Agree*	*38 (20)*	*148 (80)*	186 (46)	
*Disagree*	*31 (15)*	*175 (85)*	206 (51)	
*Not sure*			11 (3)	

	**Reason for not taking therapy**			

*Implied missed therapy*				0.001*
*Gave reason for not taking*	*50 (24)*	*156 (76)*	206 (51)	
*Never failed*	*22 (11)*	*175 (89)*	197 (49)	

*Reason for not taking medicine *				0.000*
*Being busy and forgetting*	*44 (29)*	*110 (71)*	154 (38)	
*Hiding from colleagues*	*3 (60)*	*2 (40)*	5 (2)	
*Others*	*3 (6)*	*44 (94)*	47 (11)	

*Statistically significant at level p < 0.05 by chi-square test

Majority respondents 281 (70%) had a CD4 count of more than 200 cells/ml, 280 (80%) had more than 1 year experience with ART and 138(43%) had not had side effects. ART knowledge was extremely high (100), but only 197 (49%) stated condom use as important, (82%) reported adherence and (68%) nutrition.

Most 321(80%) patients lived with family, 329(82%) had disclosed their HIV status and 282(79%) reported getting social support. Sixty nine (17%) patients found therapy disruptive of their life while 186 (46%) were embarrassed taking ARV. Most respondents 154 (38%) reported being busy and forgetting as the reason for missing therapy, while 197 (49%) had never failed to take medicine.

### Adherence to HAART

Seventy two (18%) respondents were non-adherence based on CASE adherence method, while74 (18%) reported missing at least one dose of therapy per week (< 95% adherence) (Table 2). Adherence to HAART among respondents differed significantly at *p-value *< 0.05 by age (*p = *0.017), difficulty fitting therapy in own daily schedule (*p = *0.006), social support (*p = *0.015), period on therapy (*p = *0.002), self report missed therapy (*p = *0.001), proximity to clinic where respondents refilled (*p = *0.003) and time spent at clinic per visit (*p = *0.000) by univariate analysis (Table 1). However, only having difficult fitting therapy in own schedule (OR = 2.310, CI._95 _= 1.211-4.408, *p = *0.011) and proximity to clinic where respondents refilled (OR = 2.387, CI._95 _= 1.155-4.931, *p = *0.019) predicted non-adherence by multivariate analysis (Table 3). Main reason for missing therapy was being busy and forgetting.

**Table 2:** Prevalence of non-adherence to HAART among respondents

	Adherent?	
**Methods of determining adherence**	**Yes; *n (%)***	**No; *n (%)***

a)CASE adherence Index; i.e. ≤ 10 (non-adherent)	72 (18)	331 (82)

b)missed at least once a week method (< 95 adherence)	74 (18)	329 (82)

**Table 3:** Predictors of Non-adherence to HAART among respondents

	Crude		Adjusted	
**Characteristics**	**OR (CI._95_)**	**p-value**	**OR (CI._95_)**	**p-value**

Proximity to clinic where refilled	2.740 (1.382,5.434)	0.003*	2.387 (1.155,4.931)	0.019**

Reported difficult fitting therapy in own schedule	2.282 (1.250-4.169)	0.006*	2.310 (1.211-4.408)	0.011**

Gave reasons for skipping doses	2.550 (1.477-4.401)	0.001*	2.264 (1.261-4.064)	0.006**

**Statistically significant at level p < 0.05 by multivariate analysis

## Discussion

The current study assessed non-adherence and factors associated with it in Nairobi, Kenya. Prevalence of non-adherence found in this study (18%) is comparable to the continental prevalence (23%) for Africa [1], 21% in Southwest Ethiopia [2] and 22% in Cote d'Ivore [3]. But, inconsistent with the findings of Ellis *et al*. in Kibera, Nairobi (48%) [12]; Talam *et al*. in Eldoret (56.8%) [13] and Munyao *et al*. in Mombasa [14]. The inconsistency with the findings of Eldoret study was attributed to differences in assessment methods, and inconsistency with the findings of Kibera and Mombasa studies to difference in treatment periods (2005) when ART knowledge among patients and clinicians was low [17].

In this study, younger respondents below the mean age (39.7 years) were more likely not to adhere to HAART although age did not predict. The finding is consistent with the findings of Iliyasu *et al *in Nigeria [6], Carballo *et al *in Spain [9] and Orrell *et al *in South Africa [7] but, inconsistent with the finding of Talam *et al *in Eldoret [13]. The inconsistent with Eldoret study can be attributed to difference in mean age; respondents in the Eldoret study were younger (mean age 36.1 years). Better adherence among older adults may be explained by survivor effect in that, individuals who maintain greater compliance with treatment recommendations may actually outlive those who are non-adherent. Proportion of female in this study was two-fold that of male and is similar to the proportion of HIV/AIDS reported in Kenya [18]. However, gender did not predict non-adherence to HAART in Nairobi. These findings correlated with findings of other studies [5,6,13,19]. Marital status did not predict non-adherence. Other studies have reported mixed results; Byakika *et al *found an association between marital status and non-adherence to ARV therapy in Uganda [5] while, Weiser *et al *in Botswana did not [19].

Socio-economic factors did not significantly influence adherence in this study. Findings with respect to income were consistent with the findings of Orrell *et al *in South Africa [7] but inconsistent with findings of Byakika *et al *in Uganda where patients paid for ART [5]. Finding about formal education was consistent with the findings of Weiser *et al *in Botswana [19] but, inconsistent with the findings of Carballo *et al *in Spain [9] where understanding of treatment regimen was poor [10]. Although respondents living in smaller houses were more likely not to adhere to therapy than respondents living in bigger houses, house size did not predict non-adherence. These findings are consistent with findings in other studies [9,20].

In this study, proximity to clinic predicted non-adherence. Respondents who accessed therapy in clinics within a walking distance from their homes were about two and a half times more likely not to adhere than patients who refilled in far away clinics. These findings together with social stigma associated with ART use suggested that most respondents who accessed free therapy in clinics within walking distance to their homes did so due to lack of choice; speculatively, could not afford transport cost to alternative HAART clinics. Where respondents paid for transport, amounts paid did not significantly influence non-adherence to HAART which was consistent with the finding of Byakika *et al *in Uganda [5]. Most respondents reported felt social support from friends and relatives, and it was therefore hypothesized that these friends and relatives also provided respondents with material support, making it possible to overcome cost barrier that has been associated with non-adherence to therapy in other studies [5]. Such "helpers" have been found to make their expectations for the patients to adherence known, thus, creating a responsibility on the part of patients who consequently, adhered to therapy to promote goodwill with the helpers [21]. Recipients who reported receiving social support were two folds more likely to adhere to therapy than those who did not. These findings are consistent with many ARV therapy adherence studies [16,2,22-25].

Adherence to therapy in this study increased with duration on HAART although did not predict it. This is consistent with findings of Mannheimer *et al *[15] but, inconsistent with the findings of Byakika *et al *in Uganda [5]. The inconsistency was attributed to the shorter experience with ARVs and paying for therapy in the Uganda study. Where drugs are paid for, many studies have found shortage of drugs due to economic barriers, as the most common reason for non adherence. The reported adverse effects did not significantly influence non-adherence which was consistent with findings of Weiser *et al *in Botswana [19], and Aspeling and Van Wyk in South Africa [22]. The findings could be attributed to high ART knowledge and awareness found among respondents in line with the South Africa study in which informed HIV patients tolerated side effects and adhered to therapy [22].

In this study, respondents regarded adherence to HAART extremely important to HIV treatment and translated this knowledge into positive belief about necessity of HIV medication almost universally. Belief in the benefit of therapy found together with availability of free HAART is credited for improved adherence among the respondents. This finding correlates with findings of Aspeling and Van Wyk in South Africa where adequate pre-therapy counselling and HIV education impacted adherence positively [22]. However, giving reason for skipping therapy predicted non-adherence. Three quarters of the respondents who gave reasons for skipping therapy stated being busy and forgetting as the reason and a third of them did not adhere. This finding is consistent with findings of Nieuwkerk *et al *[26] and Byakika *et al *in Uganda [5]. Having difficult fitting therapy in own daily schedule predicted non-adherence. Respondents who reported difficult with therapy were more than two folds more likely not to adhere to ARV therapy. This was consistent with the findings of Munro *et al *[8]. It was speculated, in line with Vervoort *et al *[24] finding that when medication scheme did not fit in a patient's routine, it caused them to forget to take medications and consequently resulted in poor adherence.

## Conclusion

Given the complex array of factors associated with non-adherence, no single strategy is likely to be effective for every patient. It is recommended that patients be targeted with comprehensive individualized interventions employing behavioral educational strategies to improve ability to fit therapy in own lifestyle and cue-dose training to impact forgetfulness. Further research is recommended to explain poor adherence among patients accessing therapy from ARV clinics within walking distance from their homes.

## List of abbreviations

AOR: Adjusted Odds Ratio; ART: Antiretroviral Therapy; ARV: Antiretroviral; CCC: Comprehensive Care Centre; COR: Crude Odds Ratio; HAART: Highly Active Antiretroviral Therapy; HIV: Human Immunodeficiency Virus; OR: Odds Ratio.

## Competing interests

The authors declare that they have no competing interests.

## Authors' contributions

SNW conceived, designed and conducted study, analysed data and interpreted findings. ZWN and GGM supervised and guided the student. All authors contributed to the final report and approved the final manuscript.
